# Anti-Melanogenic Effects of Umbelliferone: In Vitro and Clinical Studies

**DOI:** 10.3390/molecules29235571

**Published:** 2024-11-25

**Authors:** Da Jung Kim, Min Sook Jung, Hee Un Jin, Mi Sun Kim, Chae Eun An

**Affiliations:** Skin Science Research Center, NewLife BST Co., Ltd., Seoul 08594, Republic of Korea; djkim@newlifegroup.com (D.J.K.);

**Keywords:** umbelliferone, melanogenesis, tyrosinase, clinical trial, cosmetics

## Abstract

Melanin overexpression causes skin hyperpigmentation, which is associated with various skin disorders and cosmetic concerns. Umbelliferone, a natural coumarin found widely in plant species, has been noted for its antioxidant and anti-inflammatory effects but has received little attention for its impact on melanogenesis. Here, the effects of umbelliferone on melanogenesis were investigated in vitro and in clinical studies. The results showed that umbelliferone was non-cytotoxic to human skin and B16F10 melanoma cells. It also exhibited significant anti-melanogenic effects, reducing both melanin production and tyrosinase activity in a dose-dependent manner. This effect was achieved through a decrease in tyrosinase mRNA levels. Furthermore, umbelliferone in a formulation was stable under different temperature conditions, and after four weeks of topical application, it significantly decreased the melanin index and increased skin lightness (L*) values compared to those at the baseline. Overall, these findings demonstrate the potential of umbelliferone as a promising skin-lightening agent in the cosmetics industry.

## 1. Introduction

The skin serves as the outermost barrier between the internal and external environments and consists of the epidermis, dermis, and subcutaneous layers [1]. Within the epidermis, melanocytes play a vital role in determining skin color via melanin production [2]. This process, known as melanogenesis, acts as an important defense mechanism against external factors. Moreover, it maintains body temperature and protects the skin from ultraviolet radiation [3]. However, excessive melanin production can result in hyperpigmentation, leading to conditions such as melanoma, freckles, or age spots [4,5]. Therefore, regulating melanogenesis is essential for both the pharmaceutical and cosmetics industries [3]. Melanin is enzymatically synthesized by the tyrosinase gene family, which induces the expression of microphthalmia-associated transcription factor (MITF), tyrosinase, tyrosinase-related protein 1 (TRP-1), and tyrosinase-related protein 2 (TRP-2) [6]. Tyrosinase is a key enzyme that facilitates melanin production by converting tyrosine to dihydroxyphenylalanine (DOPA) [7].

Tyrosinase is a crucial target for melanogenesis inhibition. Various tyrosinase inhibitors, including kojic acid, hydroquinone, and arbutin, are used in pharmaceuticals and cosmetics to reduce melanin production; however, these inhibitors can lead to undesirable side effects, such as contact dermatitis, skin irritation, and transient erythema [8,9,10]. Therefore, several studies are underway to identify ingredients that offer effective anti-melanogenic properties with minimal side effects.

Coumarins are prevalent in various plant species and exhibit diverse pharmacological effects [11]. Among these, umbelliferone, also known as 7-hydroxycoumarin, hydrangine, or skimmetine, has attracted significant interest from researchers in various pharmaceutical fields. Umbelliferone (MW of 162.133 g·mol^−1^) is a secondary metabolite found in the flowers, fruits, and roots of various higher plants such as the snow lotus, carrot, and master [12]. This compound appears as yellowish-white needle-like crystals with the ability to effectively absorb ultraviolet light [13,14]. It has also shown promise in treating various conditions, including diabetes, neurodegenerative diseases, cardiovascular issues, various cancers, inflammatory disorders, and microbial infections [12,15,16,17]. A previous study indicated that this compound exhibited particularly notable cytotoxic effects against MKN-45 human gastric adenocarcinoma and MIA PaCa-2 human pancreatic cancer cells after 72 h of treatment, while demonstrating minimal cytotoxicity in NIH/3T3 fibroblasts [18]. Furthermore, when administered orally, umbelliferone at a concentration of 200 mg/kg alleviated symptoms of atopic dermatitis in mice by effectively suppressing pro-inflammatory cytokines and chemokines without causing oral toxicity [13,19,20]. Additionally, the antioxidant properties of umbelliferone are associated with its ability to scavenge free radicals and inhibit lipid peroxidation. This antioxidant effect may be attributed to the activation of the Nrf2 signaling pathway, which enhances the activity of various endogenous antioxidants, including superoxide dismutase, glutathione, catalase, and NAD(P)H-quinone oxidoreductase [21]. Thus, the pharmaceutical activity of umbelliferone suggests that it may be valuable in cosmeceuticals; however, its use in cosmetics remains to be thoroughly investigated.

Therefore, this study aimed to explore the inhibitory effects of umbelliferone on melanogenesis, both in vitro and in a clinical trial. We examined the effects of umbelliferone on tyrosinase activity and melanin production in B16F10 melanoma cells. Furthermore, we conducted a clinical trial to assess the effects of an umbelliferone-containing formulation.

## 2. Results and Discussion

### 2.1. Cell Viability and Cytotoxicity Effects of Umbelliferone

**Figure 1:**
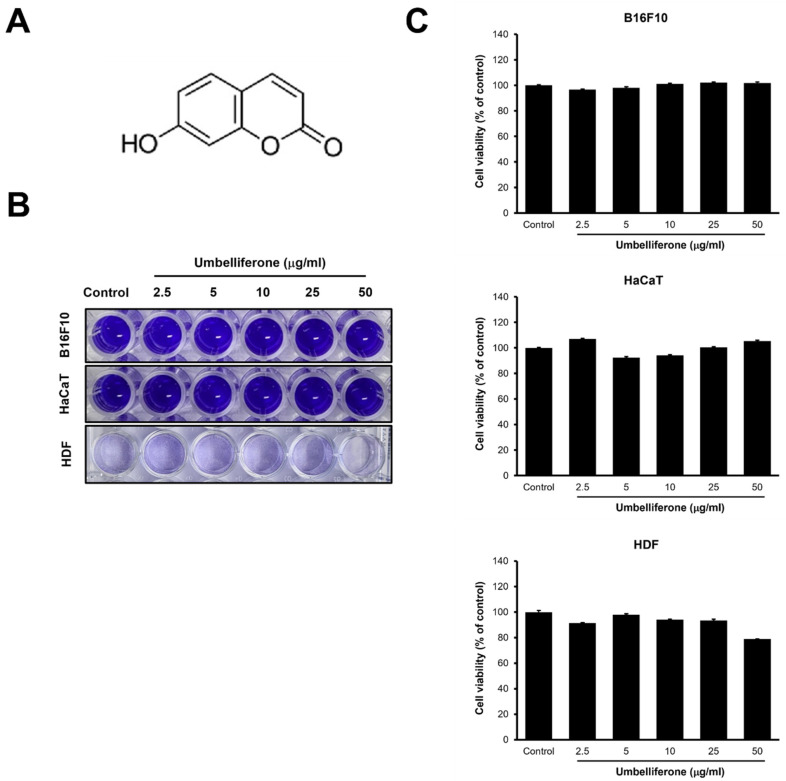
Chemical structure and cytotoxicity of umbelliferone. (**A**) Chemical structure of umbelliferone. (**B**) Crystal violet staining. B16F10 cells, HaCaT cells, and human dermal fibroblasts (HDFs) were treated with 2.5, 5, 10, 25, and 50 μg/mL of umbelliferone for 48 h. The cells were fixed and subjected to crystal violet staining. (**C**) Quantitative analysis of the crystal violet-stained cells. The stained cells from (**B**) were dissolved in 1% sodium dodecyl sulfate, and absorbance was measured at 590 nm. Each bar represents the mean ± SD from three independent experiments.

To assess the impact of umbelliferone on cell viability, B16F10 melanoma cells, HaCaT cells, and human dermal fibroblasts (HDFs) were exposed to various concentrations of umbelliferone (2.5, 5, 10, 25, 50 µg/mL) for 48 h. As shown in Figure 1, viability remained greater than 90% across all concentrations (2.5–50 µg/mL) of umbelliferone in B16F10 melanoma and HaCaT cells. However, in HDFs, cell viability dropped to below 80% at the highest umbelliferone concentration of 50 µg/mL. These results indicate that umbelliferone exhibits minimal cytotoxicity at concentrations below 50 µg/mL. Therefore, to evaluate the anti-melanogenic effects of umbelliferone, concentrations less than 50 µg/mL were used for the subsequent experiments.

### 2.2. Effect of Umbelliferone on the Extracellular and Intracellular Melanin Content in B16F10 Melanoma Cells

**Figure 2:**
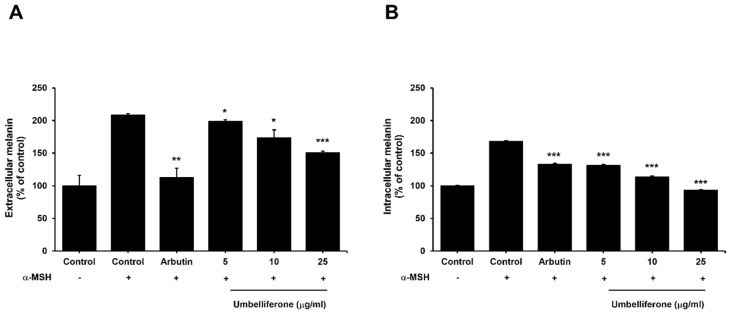
Effect of umbelliferone on melanin content. B16F10 melanoma cells were exposed to 100 nM of α-MSH with or without arbutin (1 mM) and umbelliferone (0, 5, 10, 25 μg/mL). Levels of both extracellular (**A**) and intracellular (**B**) melanin were quantified using optical density measurements at 490 nm. Each bar represents the mean ± SD of three independent experiments. * *p* < 0.05, ** *p* < 0.01 and *** *p* < 0.001 indicate significant differences compared with the α-MSH-treated control group.

To explore the impact of umbelliferone on melanogenesis regulation in α-MSH-treated B16F10 melanoma cells, the levels of both extracellular and intracellular melanin were assessed after 48 h of treatment with umbelliferone or arbutin, a well-established skin-whitening agent known for its ability to inhibit melanogenesis via tyrosinase regulation [22]. Figure 2 illustrates the inhibitory effects of umbelliferone on both extracellular and intracellular melanin production. As shown in Figure 2, umbelliferone dose-dependently reduced the levels of extracellular and intracellular melanin induced by α-MSH stimulation in B16F10 melanoma cells. These findings suggest that umbelliferone effectively suppresses melanin production in α-MSH-stimulated B16F10 melanoma cells.

### 2.3. Effect of Umbelliferone on the Tyrosinase Enzyme in the Mushroom Tyrosinase Assay

**Figure 3:**
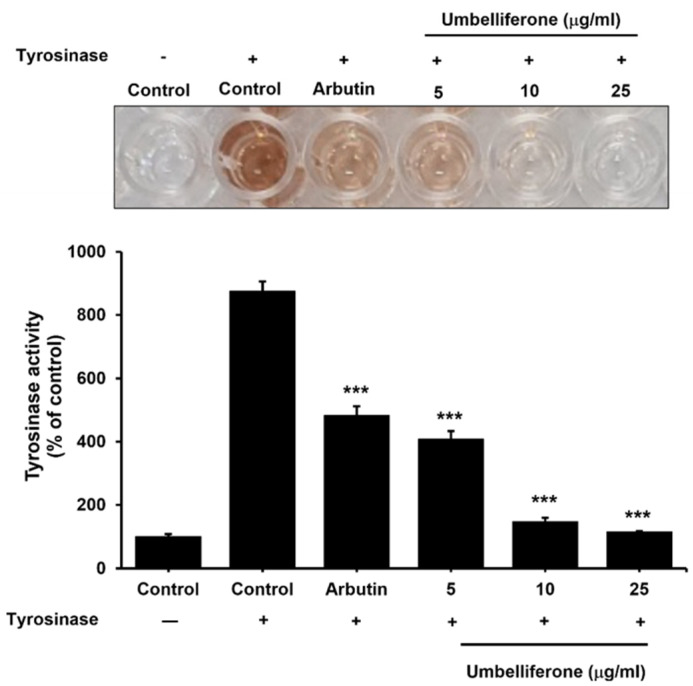
Effects of umbelliferone on tyrosinase activity. The mushroom tyrosinase solution was treated with arbutin (200 μM) and umbelliferone (0, 5, 10, 25 µg/mL), and melanin content was measured based on optical density at 475 nm. Each bar represents the mean ± SD of three independent experiments. Statistical analysis revealed a significant difference at *** *p* < 0.001 compared to the negative control with the mushroom tyrosinase solution.

As shown in Figure 3, both arbutin and umbelliferone effectively inhibited tyrosinase activity in mushroom tyrosinase-treated solutions, indicating their potential as anti-melanogenic agents. In the negative control group containing mushroom tyrosinase solution, tyrosinase activity was approximately 8.75-fold higher than that in the negative control group without mushroom tyrosinase solution. However, arbutin treatment significantly reduced tyrosinase activity by 1.8-fold compared to that in the negative control group with the mushroom tyrosinase solution. Similarly, umbelliferone treatment induced a significant dose-dependent decrease in tyrosinase activity. These findings suggest that umbelliferone inhibits melanogenesis by inhibiting tyrosinase activity.

### 2.4. Effect of Umbelliferone on Tyrosinase and MITF Expression in B16F10 Cells

**Figure 4:**
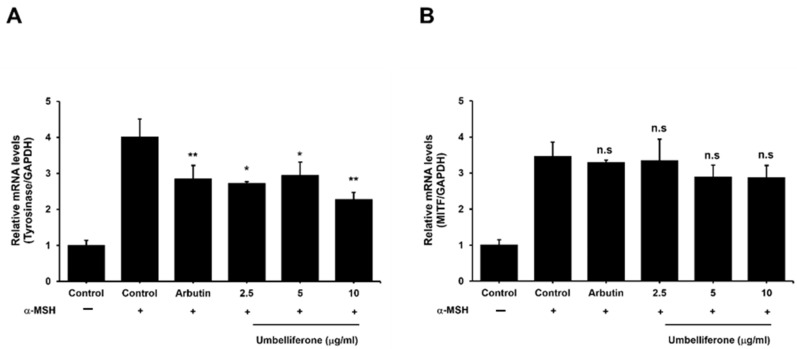
Effects of umbelliferone on mRNA expression. B16F10 melanoma cells were treated with 100 nM α-MSH alone or in combination with arbutin (200 μM) and umbelliferone (2.5, 5, 10 μg/mL). Quantitative real-time PCR was used to measure the expression levels of tyrosinase (**A**) and MITF (**B**). Each bar represents the mean ± SD of three independent experiments, with significance differences denoted by * *p* < 0.05 and ** *p* < 0.01 compared to the α-MSH-treated control group. n.s. indicates not significant.

**Figure 5:**
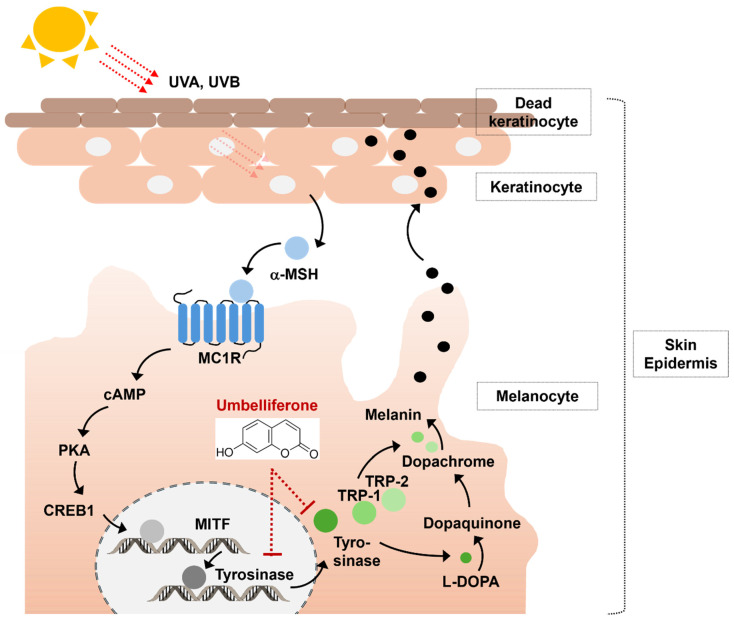
Anti-melanogenic mechanism of umbelliferone. Umbelliferone can inhibit melanin production via two mechanisms: (1) by blocking the expression of tyrosinase mRNA, and (2) by decreasing tyrosinase activation.

To determine the ability of umbelliferone to decrease tyrosinase protein levels in B16F10 melanoma cells, we investigated its effects on tyrosinase and MITF mRNA expression. Melanogenesis is controlled by genes related to this process, such as tyrosinase, TRP-1, and TRP-2, which are regulated by MITF [23]. MITF influences these genes by binding to their promoter regions [6]. To further elucidate the anti-melanogenic effects of umbelliferone, we examined its effects on tyrosinase and MITF gene expression. As shown in Figure 4A, the control group treated with α-MSH showed a fourfold increase in tyrosinase expression compared to that in the negative control. Treatment with umbelliferone at the specified concentrations significantly reduced tyrosinase expression, achieving the most notable decrease at a rate of 1.7-fold. Similarly, arbutin, known for its tyrosinase inhibitory properties, decreased tyrosinase expression by approximately 1.2-fold compared to that in the α-MSH-treated control group. However, as shown in Figure 4B, umbelliferone treatment did not lead to statistically significant changes in MITF expression levels. These findings strongly indicate that umbelliferone modulates melanogenesis primarily by suppressing tyrosinase expression without affecting MITF gene expression (Figure 5).

Tyrosinase expression can be influenced by various factors interacting with its promoter independently of MITF. For example, transcriptional co-activators like CBP and p300 bind to the N-terminal transactivation domain of MITF, thereby enhancing MITF-dependent transcription [24,25]. Likewise, lymphoid enhancer-binding factor 1 binds to the tyrosinase promoter, boosting MITF-dependent tyrosinase gene expression [26,27]. Furthermore, certain factors affect protein expression without altering mRNA levels during melanin synthesis. For example, normal human melanocytes increase the protein levels of TRP-1 and TRP-2 in response to α-MSH, even though their mRNA levels remain unchanged [28]. Similarly, glyceollin from soybeans does not affect TRP-1 and MITF mRNA levels, despite inhibiting their protein expression [29].

Based on these studies, we inferred that umbelliferone may influence the expression of proteins related to melanogenesis, including MITF. However, additional studies on the effects of umbelliferone on protein expression are necessary to validate this hypothesis.

### 2.5. Stability of Umbelliferone in Formulation Under Different Conditions

**Figure 6:**
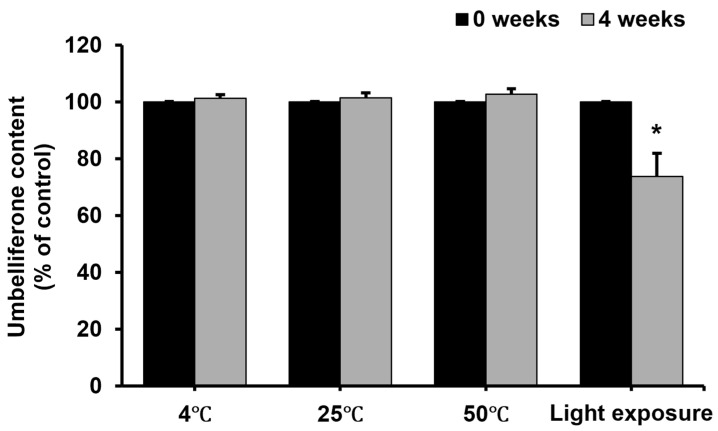
Stability of umbelliferone in a formulation at different temperatures and under light exposure. Stability of umbelliferone in a formulation at various temperatures (4, 25, and 50 °C) and under light exposure for 4 weeks. Umbelliferone content was quantified using a high-performance liquid chromatograph equipped with a C18 column. Each bar represents the mean ± SD of two independent experiments. Statistical analysis revealed a significant difference at * *p* < 0.05 compared to the untreated sample.

The changes in umbelliferone stability in a formulation at various temperatures and under sunlight exposure are shown in Figure 6. Umbelliferone is known for its instability [30,31,32], and can be converted to hydroxylated (esculetin), glycosylated (skimmin), and methylated (herniarin) derivatives during enzymatic degradation [12], potentially reducing its anti-melanogenic effect. Cosmetic manufacturing often involves high temperatures and varying pH levels, which can affect umbelliferone stability. In this study, storage at 4 °C, 25 °C, and 50 °C had no significant effect on the umbelliferone contents in the formulation for up to 4 weeks. In contrast, a significant difference was observed in umbelliferone content with sunlight exposure. Upon exposure to sunlight, the amount of residual umbelliferone decreased to approximately 73%, indicating reduced stability under light exposure. Generally, coumarins like umbelliferone are prone to light-induced reactions, such as photooxidation [33,34]. Photooxidation can occur through electrons, leading to hydrated electrons in aqueous environments, or via excited-state electron- or proton-transfer reactions [35]. Therefore, photooxidation likely affects the stability of umbelliferone in the ampoule formulation. The reduced photostability of umbelliferone upon sunlight exposure can diminish its effectiveness in cosmetic products. Therefore, evaluating umbelliferone photostability is essential for developing cosmetics containing umbelliferone. Common strategies for enhancing photostability include protecting the product from light using appropriate primary and secondary packaging [36]. Employing liposome encapsulation and stabilizers can also improve photostability. For instance, liposome entrapment has been shown to enhance the photostability of various drugs, including riboflavin [37], doxorubicin [38], vitamin A palmitate [39], and Rose Bengal [40]. Further, using stabilizers like antioxidants can improve the photostability of substances prone to photooxidation, such as umbelliferone [41,42]. Therefore, we plan to conduct further experiments to enhance the photostability of umbelliferone in cosmetic formulations.

### 2.6. Effect of Umbelliferone on Skin Pigmentation and Lightness in a Clinical Trial

**Figure 7:**
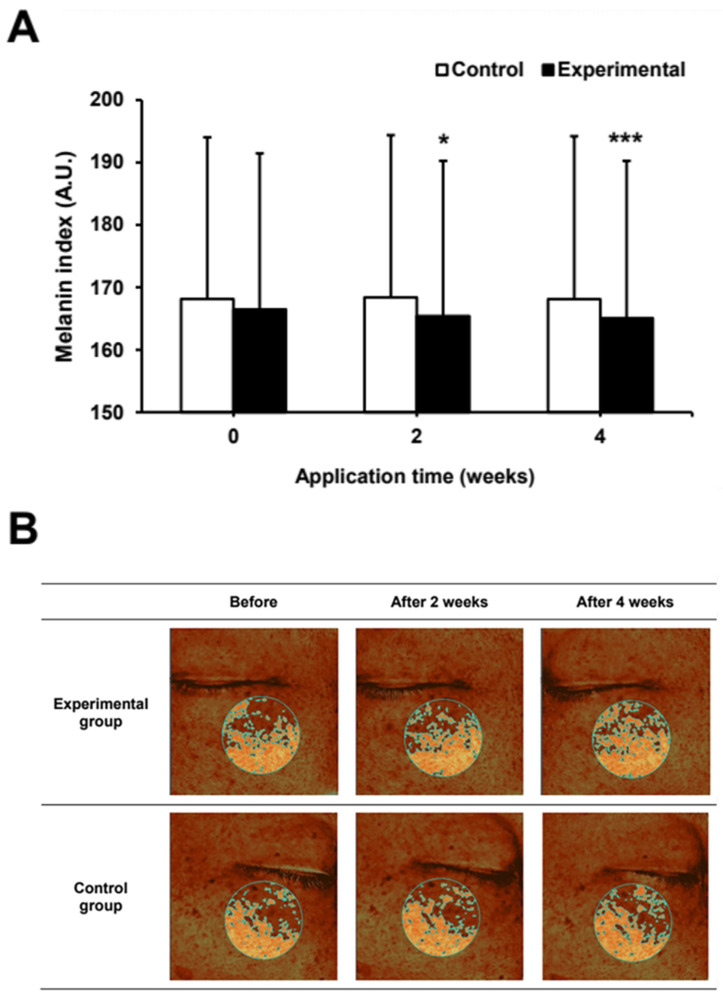
Effect of umbelliferone ampoule on skin pigmentation (**A**) Changes in the melanin index following umbelliferone application for 4 weeks. In all, 12 subjects, aged 20–59 years, participated in the study. The melanin index was analyzed at 2 and 4 weeks after applying the test ampoule (experimental group) containing 0.0025% umbelliferone, and the control ampoule (control group) without umbelliferone. Data represent the mean ± SD of three independent experiments, with statistical significance denoted as * *p* < 0.05 and *** *p* < 0.001 compared to the melanin index before application (0 weeks) of the experimental ampoule containing umbelliferone. (**B**) Representative images of skin pigmentation treated with ampoules containing umbelliferone. Antera 3D CS, a 3D skin imaging device, was used to photograph the pigmented area.

**Figure 8:**
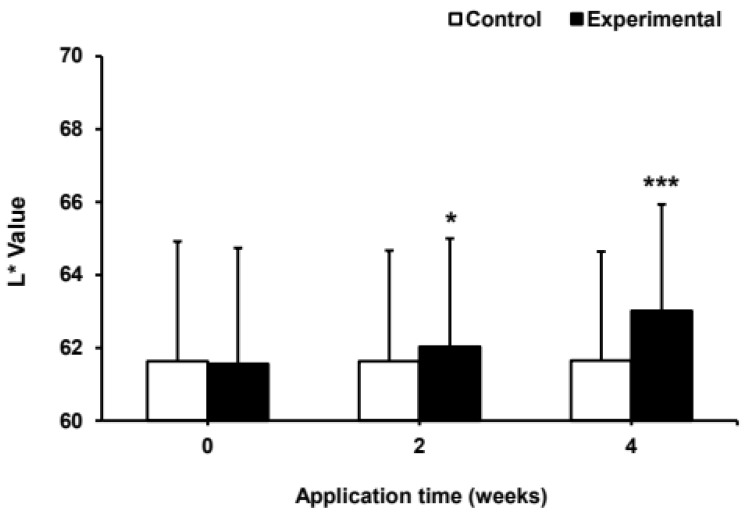
Effect of umbelliferone ampoules on skin tone. The L* value was measured at 2 and 4 weeks after applying the test ampoule (experimental group) containing 0.0025% umbelliferone, and the control ampoule (control group) without umbelliferone using spectrophotometry. Data represent the mean ± SD of three independent experiments, with statistical significance denoted as * *p* < 0.05 and *** *p* < 0.001 compared to the L* value before application (0 week) of the experimental ampoule containing umbelliferone.

Based on the in vitro results, the efficacy of umbelliferone on skin pigmentation and lightness were evaluated in 12 participants over a 4-week period. The treatments involved the application of an experimental ampoule containing 0.0025% (25 μg/mL) umbelliferone and a control ampoule without umbelliferone. In a randomized trial, the whitening ampoule with umbelliferone showed significant improvements in skin pigmentation and lightness compared to those with the control ampoule without umbelliferone. The group using the umbelliferone-containing ampoule experienced a 0.65% and 2.62% reduction in hyperpigmentation after 2 and 4 weeks, respectively, whereas the control group showed only 0.16% and 0.03% improvement at the same intervals (Figure 7). Additionally, the increase in skin lightness was 0.78% and 2.40% at 2 and 4 weeks in the umbelliferone-treated group, respectively, whereas the control group showed minimal increases of 0.02% and 0.05%, respectively (Figure 8). These results indicated that the ampoule with umbelliferone effectively decreased skin hyperpigmentation and enhanced lightness in the treated areas. No instances of skin irritation or adverse reactions were reported, indicating the safety of umbelliferone upon topical application. However, further research is required to fully understand the skin-whitening effects of umbelliferone. This study had some limitations, including (1) a small sample size and single-center recruitment, (2) absence of positive control, and (3) a short study duration; longer trials with follow-up periods exceeding 4 weeks are thus recommended.

## 3. Materials and Methods

### 3.1. Cell Culture and Chemicals

B16F10 melanoma cells, human keratinocytes (HaCaT), and human dermal fibroblasts (HDF) were obtained from the Korea Cell Line Bank (Seoul, Republic of Korea). The cells were cultured in media containing 10% fetal bovine serum (Gibco, Carlsbad, CA, USA) and 1% penicillin–streptomycin (Gibco), and maintained at 37 °C in a humidified atmosphere containing 5% CO_2_. All chemicals were purchased from Sigma-Aldrich (St. Louis, MO, USA), unless otherwise stated.

### 3.2. Cell Viability and Cytotoxicity

B16F10 melanoma cells, HaCaT cells, and HDFs at passage 6 were used in all experiments. To assess the potential cytotoxic effects of umbelliferone, cells were seeded in a 24-well plate at a density of 1 × 10^5^ cells/well and allowed to adhere for 24 h. Subsequently, the culture medium was aspirated and replaced with fresh medium containing umbelliferone at various concentrations (2.5, 5, 10, 25, or 50 μg/mL). After 48 h of exposure, the cells were fixed with 4% paraformaldehyde and stained with 1% crystal violet solution for 15 min. The staining solution was discarded, and the wells were washed four times with phosphate-buffered saline before air-drying the plates. Subsequently, 500 μL of 1% sodium dodecyl sulfate solution was added to each well and incubated for an additional 15 min. A 200 μL aliquot of the resulting solution was transferred to a 96-well plate to measure optical density at 590 nm using a microplate reader (Bio-Tek, Winooski, VT, USA).

### 3.3. Determination of Melanin Content

B16F10 melanoma cells were seeded in a 6-well plate at a density of 5 × 10^4^ cells/well and pre-incubated for 24 h. Prior to treatment with umbelliferone (5, 10, 25 μg/mL) and arbutin (1 mM) for 48 h, the cells were stimulated with α-MSH (100 nM) for 1 h. Arbutin was used as the positive control. To measure extracellular melanin content, the supernatants were assessed at 490 nm using a microplate reader. To determine the intracellular melanin content, cells were treated with 1 N NaOH containing 10% dimethyl sulfoxide (DMSO) at 80 °C for 1 h, and the optical density of the resulting solution was measured at 490 nm using a microplate reader.

### 3.4. Tyrosinase Activity Assay

Tyrosinase activity was assessed spectrophotometrically using a method adapted from Alam et al. [43]. Specifically, mixtures containing 0.1 M phosphate buffer (pH 6.5) (100 μL), 1 mM L-tyrosine (100 μL), and mushroom tyrosinase solution (500 units per well in phosphate buffer, pH 6.5) with or without umbelliferone at different concentrations (5, 10, 25 μg/mL) and arbutin (200 μM) were prepared in a 96-well plate. The plate was incubated at 37 °C for 15 min, and the absorbance at 475 nm was measured using a microplate reader.

### 3.5. Quantitative Real-Time Polymerase Chain Reaction

B16F10 cells were cultured in 6-well plates at a density of 1 × 10^6^ cells/well for 24 h. Following stimulation with 100 nM α-MSH for 1 h, cells were treated with either 200 μM arbutin or umbelliferone (2.5, 5, 10 μg/mL) for 48 h. Total RNA was extracted using the RNeasy Mini Kit (Qiagen, Hilden, Germany) and quantified using a microplate reader (Epoch 2; BioTek, Winooski, VT, USA). RNA was reverse transcribed into first-strand complementary DNA (cDNA) using a QuantiTect Reverse Transcription Kit (Qiagen). Each cDNA sample was amplified using PowerUp™SYBR™ Green Master Mix for quantitative PCR (qPCR) (Thermo Fisher Scientific, Waltham, MA, USA) using specific primers designed for specific cellular RNA targets (see Table 1 for details). Real-time PCR was conducted using the QuantStudio 3 system (Applied Biosystems, Foster City, CA, USA), involving 40 cycles of denaturation at 95 °C for 5 s and annealing at 60 °C for 34 s. Gene expression levels were normalized to those of GAPDH to determine the relative expression levels of each gene.

### 3.6. Formulation of the Ampoule Containing Umbelliferone

A viscous liquid ampoule containing umbelliferone was formulated in this study. The ampoule was produced at room temperature (25 °C) using a Homogenizing Disper Model 2.5 (PRIMIX speed mixer, Osaka, Japan). Initially, the aqueous-phase components (water, glycerin, polyglycerin-3, and viscosity-enhancing agents) were combined in a mixing beaker and mixed for 3 min at 2500 rpm. Subsequently, the oily phase components (fragrance, alcohol, and solubilizer) were added directly to the same beaker and mixed for an additional 3 min at 2000 rpm. For the test sample, 0.0025% (25 μg/mL) umbelliferone was added directly to the mixing cup during preparation, whereas the control sample did not contain umbelliferone.

### 3.7. Stability Test of Umbelliferone in the Formulation

To assess stability, ampoules containing 0.0025% (25 μg/mL) umbelliferone were individually subjected to different conditions: 4 °C, 25 °C, and 50 °C, and exposure to sunlight for a duration of 4 weeks. After incubation, the samples were analyzed using high-performance liquid chromatography (HPLC, Futecs, Republic of Korea) equipped with a Hector M C18 column (250 mm × 4.6 mm, particle size 5 μm, RStech, Republic of Korea). Separation was achieved using a mobile phase of methanol/acetic acid (90:10, *v*/*v*) at a flow rate of 0.5 mL/min, with both the column temperature and mobile phase maintained at 35 °C.

### 3.8. Randomized Controlled Clinical Trial

The clinical trial spanned 4 weeks and included 12 healthy Korean male and female participants aged 20–59 years, with a mean age of 26.7 ± 7.1 years. The exclusion criteria included a history of contact dermatitis or photoallergic dermatitis, prolonged use of steroid-containing skin preparations exceeding one month, and sensitive or hypersensitive skin types. This study was approved by the Institutional Review Board (IRB) of Semyung University Korean Medicine Hospital (Approval Number: GCC-040-23-001), and the trial was conducted from November to December 2023. Written informed consent was obtained from each participant before enrollment in the study.

The test samples were prepared as viscous liquid ampoules, with the experimental group containing 0.0025% umbelliferone and the control group without umbelliferone. The samples were stored in identical containers to ensure a double-blind design. Light-resistant containers were used in this study. Each participant randomly applied the test and control samples to the right and left sides of their face, respectively, without knowledge of the applied sample until the study was concluded. Participants applied the samples twice daily (morning and evening) for four weeks after washing their faces.

During each visit, participants cleansed their faces with the provided cleanser and rested in a room maintained at a constant temperature (22 ± 2 °C) and humidity (50 ± 10%) for 30 min before measurement to standardize the conditions. The evaluation included measurement of the melanin index (M-index) for the hyperpigmented skin areas using a Mexameter MX18 (Courage+ Khazaka Electronic GmbH, Köln, Germany) and skin lightness (L* value) of the cheek area at 2 and 4 weeks using a CM-26d spectrophotometer (Konica Minolta, Inc., Japan). Additionally, pigmented skin areas were imaged using an Antera 3D CS (Miravex Ltd., Dublin, Ireland).

### 3.9. Statistical Analysis

For in vitro tests, the results were presented as the mean percentage ± standard deviation (SD) from three independent experiments. Statistical significance was assessed using GraphPad Prism (La Jolla, CA, USA), with *p* < 0.05 considered statistically significant.

For clinical efficacy assessments, statistical analyses were performed using SPSS Statistics version 26.0. Repeated-measures analysis of variance (ANOVA) with Bonferroni’s correction was employed as the parametric method. Statistical significance was determined at *p* < 0.05 and *p* < 0.001.

## 4. Conclusions

Overall, our study evaluated the effects of umbelliferone on melanogenesis, both in vitro and in a clinical trial. We found that umbelliferone exhibited low cytotoxicity in the treated cells. Additionally, it effectively decreased melanin production and tyrosinase activity by regulating tyrosinase mRNA levels. Furthermore, umbelliferone was stable across various temperatures and significantly reduced skin melanin levels while enhancing skin lightness in a clinical setting. This research is crucial, as it represents the first effort to validate the effects of umbelliferone on melanogenesis. Our findings indicate that umbelliferone has significant potential as a whitening agent in cosmetic applications.

## Figures and Tables

**Table 1:** Oligonucleotide primer sequences used for quantitative real-time PCR.

Gene	Primer Sequence (5′–3′)
Tyrosinase	F: 5′-ATA GGT GCA TTG GCT TCT GG-3′
	R: 5′-TCT TCA CCA TGC TTT TGT GG-3′
MITF	F: 5′-TCA AGT TTC CAG AGA CGG GT-3′
	R: 5′-CAT CAT CAG CCT GGA ATC AA-3′
GAPDH	F: 5′-ACC CAC TCC TCC ACC TTT GA-3′
	R: 5′-CTG TTG CTG TAG CCA AAT TCG T-3′

## Data Availability

Data are contained within the article.
